# CAREER COMPETENCE IN LATE LIFE WORK

**DOI:** 10.1093/geroni/igad104.1104

**Published:** 2023-12-21

**Authors:** Lyn Holley

**Affiliations:** University of Nebraska Omaha, Omaha, Nebraska, United States

## Abstract

Career Competence in Late Life Work For most professionals, ageing manifests gradually – perhaps a practically imperceptible “slowing” of thought occurs sooner and sooner in the semester, perhaps a stiffness in joints or dozens of other cues from our gerontology lectures and texts; “natural” phenomena that signal we should be thinking of retirement. Self perceptions accumulate, their weight discouraging attempts to continue to teach energetically and creatively. Post-docs, pre-tenure faculty, and financially-challenged Deans withdraw, and the “voice in your head” agrees you are not what you were and should turn to figuring out how you can best spend the next ten to thirty years as your connectedness and career competence continue to dwindle. Meanwhile, the need for experienced faculty expands, and support to fund unemployed older adults edges nearer to financial collapse. A review of the literature on these phenomena suggests that excising the embedded ageist stereotypes is likely to unleash and focus energy on continuing to learn and contribute to society. Many colleges already have begun to provide support for late life learning, and to design opportunities for retirees and other older adults to contribute to the higher learning enterprise that enhance rather than shrink available “slots”, and expand capacity for research and education.

